# Transcranial Alternating Current Stimulation With Gamma Oscillations Over the Primary Motor Cortex and Cerebellar Hemisphere Improved Visuomotor Performance

**DOI:** 10.3389/fnbeh.2018.00132

**Published:** 2018-07-05

**Authors:** Shota Miyaguchi, Naofumi Otsuru, Sho Kojima, Kei Saito, Yasuto Inukai, Mitsuhiro Masaki, Hideaki Onishi

**Affiliations:** Institute for Human Movement and Medical Sciences, Niigata University of Health and Welfare, Niigata, Japan

**Keywords:** transcranial alternating current stimulation, gamma oscillations, primary motor cortex, cerebellar hemisphere, visuo-motor performance

## Abstract

Transcranial alternating current stimulation (tACS) can be used to modulate oscillatory brain activity. In this study, we investigated whether tACS applied over the primary motor cortex (M1) and cerebellar cortex region improved motor performance. We applied tACS (1.0 mA) to 20 healthy adults while they performed an isometric force task with some visuomotor control using their right index finger. Gamma (70 Hz) oscillations in the Experiment 1 or beta (20 Hz) oscillations in the Experiment 2 were applied for 30 s over the left M1, right cerebellar hemisphere or both regions (“M1-Cerebellum”), and errors performing the task were compared. Beta-oscillation tACS did not affect motor performance. With the gamma-oscillation tACS, a negative correlation was found between the difference of error in the M1-Cerebellum condition and the number of errors in the sham condition (*P* = 0.005, Pearson’s *r* = −0.597), indicating that motor performance improved with M1-Cerebellum tACS for subjects with low motor performance in the sham condition. Those who performed poorly in the sham condition made significantly fewer errors with M1-Cerebellum tACS (*P* = 0.004). Thus, for subjects with poorer motor performance, tACS with gamma oscillations applied over the M1 and contralateral cerebellar hemisphere improved their performance.

## Introduction

Oscillatory brain activity plays an important role in various brain functions, such as cognition, arousal, memory and motor system. The oscillatory brain activity is classified by five frequency bands: delta (<4 Hz), theta (4–8 Hz), alpha (8–12 Hz), beta (13–30 Hz) and gamma (>30 Hz). In the primary motor cortex (M1), beta band activity has been shown to decrease prior to and during voluntary movement (Muthukumaraswamy, 2011) and to increase during isometric contraction (Brown and Marsden, 1998), whereas gamma band activity increases prior to and during motor performance (Hamada et al., 1999; Muthukumaraswamy, 2011) and also affects the motor response time (Shibata et al., 1999). Taken together, these findings indicated that beta band activity is antikinetic activity on motor performance, whereas gamma band activity is prokinetic in nature. Recent research has therefore focused on artificially modulating oscillatory activity in the gamma bands in the motor-related area of the brain to improve motor performance.

Transcranial alternating current stimulation (tACS) is a noninvasive method of brain stimulation that can modulate oscillatory brain activity in the cortical region (Abd Hamid et al., 2015; Antal and Herrmann, 2016). By applying alternating current through two electrodes attached to a subject’s head, it is possible to entrain the oscillation of the cortex directly under one electrode to a specific frequency (Helfrich et al., 2014a, b; Naro et al., 2016). Previous studies have demonstrated that applying tACS over M1 resulted in attenuated finger movement velocity (Pogosyan et al., 2009) and force (Joundi et al., 2012) at beta band frequencies (“beta tACS”), whereas it increased finger movement velocity (Moisa et al., 2016) and force (Joundi et al., 2012) at gamma band frequencies (“gamma tACS”). On the other hand, there is no generally accepted interpretation of the effect of tACS over M1 on the motor performance of tasks requiring motor control, such as a visuomotor tracking task. It has been reported that although beta and gamma tACS on the M1 and shoulder had no effect on the visuomotor tracking task (Moisa et al., 2016), tACS at 80 Hz on the M1 and Cz areas is also reported to improve the performance of a visuomotor tracking task during stimulation (Santarnecchi et al., 2017). These conflicting results might involve differences in the electrode positions; nevertheless, this has not been examined. However, previous studies have demonstrated that gamma band activity also occurs in the cerebellum (de Solages et al., 2008; Cheron and Cheron, 2018), and it plays a role in synchronization of the sensory and motor cortices (Popa et al., 2013). It has been reported that activity of the cerebellar cortex region is also important for movement tasks requiring motor control (Ehsani et al., 2016; Spampinato et al., 2017); if the neural network between M1 and the cerebellar cortex region does not function properly, exercise cannot be performed efficiently and smoothly. These reports suggest that the outcome of a movement task requiring motor control may be improved by modulating the activity of both M1 and the cerebellar cortex region rather than modulating M1 activity alone. A “binding theory” has been proposed, in which neural populations excited in different cortical regions synchronize with the gamma band oscillation, strengthening the intercortical neural network (Lee et al., 2003). Previous studies have reported that the neural activities of the primary and secondary somatosensory cortex are synchronized in the gamma band in the perceptual process (Hagiwara et al., 2010), and bilateral M1 are synchronized in the gamma band in the bilateral handed motor tasks (Minc et al., 2010). In other words, the gamma synchrony plays an important role in the binding among the cortical areas involved in complex motor task, perception and memory (Hagiwara et al., 2010; Minc et al., 2010; Yamamoto et al., 2014). tACS can modulate the neural connectivity in the cortical area under the electrodes (Helfrich et al., 2014b; Bächinger et al., 2017). Therefore, gamma tACS over M1 and the cerebellar cortex region might strengthen the intercortical neural network between M1 and the cerebellar cortex region. We therefore hypothesized that strengthening the neural network between the cortices by gamma tACS over M1 and the cerebellar cortex region may improve performance of a movement task requiring motor control. Thus, the aim of the present study was to clarify whether motor performance improves with gamma tACS over M1 and the cerebellar cortex region. In the present study, we examined the change in motor performance of an isometric force task with some visuomotor control during gamma (70 Hz) or beta (20 Hz) tACS over M1 and the cerebellar cortex region. We hypothesized that the motor performance is improved by gamma tACS over M1 and the cerebellar cortex region and that the motor performance does not change in beta tACS.

## Materials and Methods

We conducted two experiments to clarify whether motor performance was improved by gamma tACS over M1 and the cerebellar cortex region. Both involved the same subjects and followed the same protocol for testing motor performance in an isometric force task with some visuomotor control. In Experiment 1, we analyzed performance during gamma tACS. In Experiment 2, which took place after an interval of at least 2 months, we analyzed the performance during beta tACS to verify the specificity of the stimulation frequency.

### Subjects

Twenty healthy men, aged 21.5 ± 1.7 years (mean ± standard deviation), participated in both experiments. All were right-handed, were taking no medication and had no central nervous disease, psychiatric disorder, or orthopedic disease. The study followed the recommendations of the ethics committee of Niigata University of Health and Welfare, who approved the protocol, and was conducted in accordance with the principles of the Declaration of Helsinki, with written informed consent obtained from all the subjects.

### Transcranial Alternating Current Stimulation

**Figure 1:**
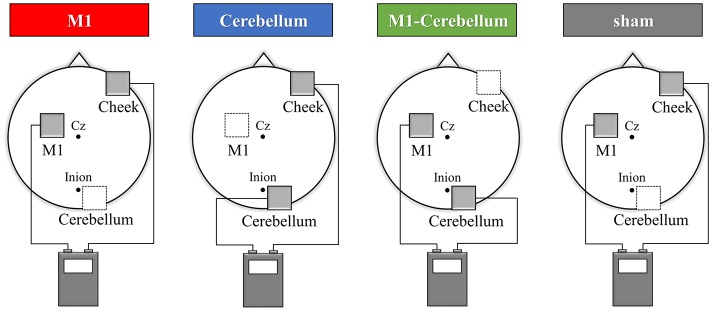
tACS electrode placement in Experiment 1. The electrodes were placed on the scalp over the left M1, right cerebellar cortex region and right cheek. The gray squares indicate the active electrodes and white squares indicate unstimulated electrodes. By switching the hardware plugs, an alternating current was applied using two of the three electrodes. Four stimulation conditions were applied: (1) pseudo-stimulation of the left M1 and right cheek (sham condition), (2) stimulation of the left M1 and right cheek (M1 condition), (3) stimulation of the right cerebellar cortex region and right cheek (Cerebellum condition) and (4) stimulation of the left M1 and right cerebellar cortex region (M1-Cerebellum condition). tACS, transcranial alternating current stimulation; M1, primary motor cortex.

tACS was delivered using a DC stimulator (Eldith, neuroConn GmbH, Ilmenau, Germany) through two saline-soaked surface sponge electrodes (5 × 5 cm, 25 cm^2^) of three electrodes. The electrodes were placed on the scalp over the left M1, right cerebellar cortex region and right cheek. By switching the hardware plugs, an alternating current was applied using two of the three electrodes. The center of the M1 electrode was placed on the scalp over the left M1 hot spot, defined as the position where magnetic stimulation to the left M1 consistently resulted in the largest motor evoked potential of the right first dorsal interosseous, as assessed using a magnetic stimulator (Magstim, Whitland, UK) and figure-of-eight coil (diameter, 9.5 cm). The center of the right cerebellar cortex electrode was placed 2.0 cm below and 3.0 cm laterally to the inion (Naro et al., 2017). The reference electrode was placed the right cheek to minimize any unintended effect of other cortex (Im et al., 2012; Tseng et al., 2018). tACS was then applied at 70 Hz or 20 Hz for Experiments 1 and 2, respectively, at an intensity of 1.0 mA (peak to peak), with a fade in/out of 5 s and a duration of 30 s (Moisa et al., 2016). It has been shown that this stimulation condition probably does not modulate cortical excitability (Moliadze et al., 2010). Four stimulation conditions were applied: (1) pseudo-stimulation of the left M1 and right cheek (sham condition), (2) stimulation of the left M1 and right cheek (M1 condition), (3) stimulation of the right cerebellar cortex region and right cheek (Cerebellum condition) and (4) stimulation of the left M1 and right cerebellar cortex region (M1-Cerebellum condition; Figure 1). In the sham condition, stimulation was performed only for 10 s of fade in/out. tACS application in this study complied with recent safety guidelines (Antal et al., 2017).

### Evaluation of Motor Performance

**Figure 2:**
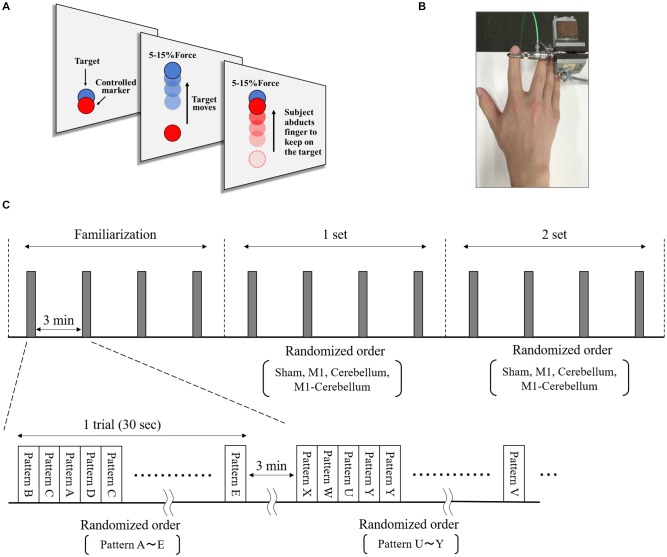
The isometric force task with some visuomotor control. **(A)** The subjects tracked the vertical movements of the blue target marker as accurately as possible by moving a red control marker up and down using abduction force of their index finger. **(B)** The tensiometer was fixed to his right index finger. **(C)** We created 25 movement patterns (Patterns A to Y) that randomly combined five frequencies and five movement ranges, and then randomly selected five movement patterns that did not overlap in frequency or movement range. For each trial, the target was set to move through these five selected movement patterns three times in a random order over 30 s. Each subject performed a total of 12 trials, separated by intervals of 3 min. The five movement patterns used for each trial were randomly selected.

The subjects’ motor performance during tACS was evaluated using an isometric force task with some visuomotor control (Figure 2A). This involved isometric abduction movement of the right index finger (Godfrey et al., 2013; Hirano et al., 2015), measured by a tensiometer (Force link 9311B, Kistler, Winterthur, Switzerland) and force control software (Niigata Prefecture Industrial Technology Research Institute, Niigata, Japan). During the experiments, the subject was comfortably seated with the right shoulder in slight abduction, the elbow in 90° flexion, and the right forearm in a pronated position. The tensiometer was fixed to his right index finger (Figure 2B). A 20-inch display connected to a notebook PC was placed at a position 60 cm in front of the subject. This displayed two markers, a moving blue marker (the target) and a red marker (the control marker) that moved up and down according to the abduction force of the subject’s index finger. The subject was instructed to apply appropriate pressure with his finger to cause the control marker to track the target marker as accurately as possible. The target was set to move up and down at five frequencies (0.4 Hz, 0.45 Hz, 0.48 Hz, 0.59 Hz and 0.67 Hz) with five ranges of movement (0%–5%, 0%–8%, 0%–10%, 0%–12% and 0%–15% of the subject’s index finger maximum abduction force). We created 25 movement patterns (Patterns A to Y) through random combinations of the five frequencies and five movement ranges, and then randomly selected five movement patterns without overlap in frequency and movement width. In each trial, the target was set to move through five randomly selected movement patterns three times in a random order. The duration of each trial was 30 s.

### Experimental Procedure

The isometric maximum abduction force of the subject’s right index finger was measured and used to set the intensity and movement pattern of the tracking task. The electrodes were then placed on the scalp, as described. The subject performed four familiarization trials, separated by intervals of 3 min, to gain sufficient practice and understanding of the task. The subject then performed four trials, again separated by intervals of 3 min, during which each of the four tACS conditions were executed in random order, followed by four more trials in which the four conditions were repeated (Figure 2C).

### Experiments 1 and 2

In Experiment 1, the tACS conditions used gamma tACS set at a frequency of 70 Hz. After at least 2 months (Hirano et al., 2015), Experiment 2 was performed with the protocol of Experiment 1, using beta tACS set at a frequency of 20 Hz. However, tACS at 16–32 Hz can elicit phosphenes (i.e., a flickering perception of light) during the stimulation (Raco et al., 2014), which could disturb the performance of the isometric force task with some visuomotor control. For Experiment 2, therefore, we conducted a preliminary experiment with four of the subjects, using the same electrode positions as in Experiment 1. All four subjects complained of strong phosphenes under the conditions in which the left M1 and right cheek, or the right cerebellum and right cheek, were stimulated. The position of the right cheek electrode was therefore changed to the left shoulder to minimize the phosphenes, which reduced their strength and appearance rate. Thus, the electrodes in Experiment 2 were placed over the left M1, right cerebellar cortex region and left shoulder. The stimulation intensity, stimulation time and fade in/out time of the tACS were the same as in Experiment 1; and, apart from the different placement of the one electrode, the same four stimulation conditions were applied: sham, M1, Cerebellum and M1-Cerebellum. To examine the effect of the phosphenes during the tracking task, the subjects rated the intensity of their phosphenes on 7-point scale (0 = “no phosphenes,” 6 = “very strong phosphenes”) (Raco et al., 2014).

### Data and Statistical Analysis

During each task, the software measured deviations in abduction force of the finger from that needed to match the movement of the target, allowing an error range of ±5% of the force. The total of the deviations for every movement of the target was used as the task error for each trial. For each tACS condition, the mean value of the task error for the two sets was used as the index of motor performance. The 20 subjects were divided into two equal groups (each *n* = 10) according to their performance for the sham condition: the low-performance subgroup, with higher task error values, and the high-performance subgroup, with lower task error values. Paired *t-test* was used to compare task error in the sham condition of Experiments 1 and 2. One-way repeated-measures ANOVAs were used to compare task errors among the tACS conditions and the intensities of the phosphenes for each tACS condition in Experiment 2. A mixed analysis of variance (ANOVA) with Group as between-subjects factor and stimulation conditions as within-subjects factor were used to compare task errors in the Experiment 1. The sphericity of the data was tested using Mauchly’s test, and Greenhouse–Geisser corrected significance values were used when sphericity was lacking. *Post hoc* analyses were performed using the Bonferroni method. The difference in task error between each tACS and sham condition was calculated (the “difference of task error”). Pearson’s correlation coefficients were calculated to investigate the correlation between the task error during the sham condition and the difference in task error. Differences were considered statistically significant at *P* < 0.05 for all analyses. IBM SPSS statistics Ver. 24 (IBM Corp., Armonk, NY, USA) was used for the statistical analysis.

## Results

### Motor Performance During Gamma tACS

**Figure 3:**
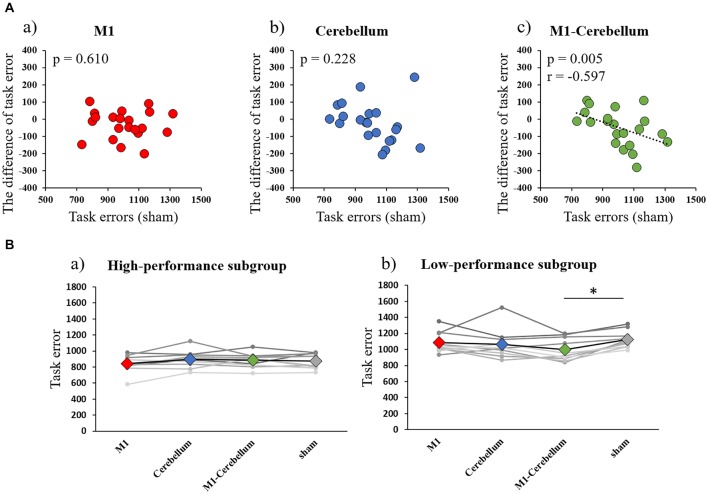
**(A)** Correlations between the difference in task error for each tACS condition and task errors during the sham condition (Experiment 1). **(a)** M1 condition. **(b)** Cerebellum condition. **(c)** M1-Cerebellum condition. **(B)** The mean values of task errors in gamma tACS for each performance subgroup (Experiment 1). **(a)** High-performance subgroup. **(b)** Low-performance subgroup. *p < 0.05. tACS, transcranial alternating current stimulation; M1, primary motor cortex.

The mean values of task errors (mean ± standard deviation) for each tACS condition were 962.6 ± 38.1 (M1 condition), 979.8 ± 38.0 (Cerebellum condition), 941.3 ± 28.9 (M1-Cerebellum condition) and 999.9 ± 36.0 (sham condition). A one-way repeated-measures ANOVA revealed no statistical difference (*F*_(3,42)_ = 2.399, *P* = 0.081, partial *η*^2^ = 0.146). A significant negative correlation was found between the difference in task error for the M1-Cerebellum condition (i.e., task error difference between M1-Cerebellum and sham) and the task error during the sham condition (*P* = 0.005, Pearson’s *r* = −0.597; Figure 3A). This indicated that, for subjects with a poorer motor performance during the sham condition, their performance improved with M1-Cerebellum tACS. No significant correlation was found for the M1 (*P* = 0.610) or Cerebellum (*P* = 0.228) conditions. The subjects were divided according to their performance under the sham condition into equally sized low- and high-performance subgroups. Table 1 shows the mean values of task error for each tACS condition and subgroups. Figure 3B shows the task error for each tACS condition for each subgroup; a mixed ANOVA revealed significant interaction (*F*_(2.483,44.693)_ = 4.508, *P* = 0.011, partial *η*^2^ = 0.200). No significant difference was found the main effect of stimulation conditions (*F*_(2.483,44.693)_ = 2.595, *P* = 0.074, partial *η*^2^ = 0.126). *Post hoc* analyses showed that the task error under the M1-Cerebellum condition was significantly less than that during the sham condition (*P* = 0.004), indicating that the low-performance subgroup improved in motor performance under the M1-Cerebellum condition. In the high-performance subgroup, the ANOVA showed no significant main effect (*F*_(3,27)_ = 1.904, *P* = 0.153, partial *η*^2^ = 0.175).

**Table 1:** The mean values of task error for each tACS condition and subgroups (mean ± SD).

		Ml	Cerebellum	Ml-Cerebellum	Sham
Gamma tACS	Low-performance subgroup	1083.9 ± 129.1	1062.8 ± 184.5	997.4 ± 141.4	1124.8 ± 106.3
	High-performance subgroup	841.4 ± 109.4	896.8 ± 107.8	885.3 ± 91.0	875.0 ± 93.4
Beta tACS	Low-performance subgroup	1081.9 ± 183.1	1071.6 ± 145.0	1072.5 ± 178.2	1093.1 ± 85.9
	High-performance subgroup	872.4 ± 116.2	859.4 ± 119.4	823.1 ± 130.9	826.7 ± 96.7
					mean ± SD

*tACS, transcranial alternating current stimulation; SD, standard deviation*.

### Motor Performance During Beta tACS

**Figure 4:**
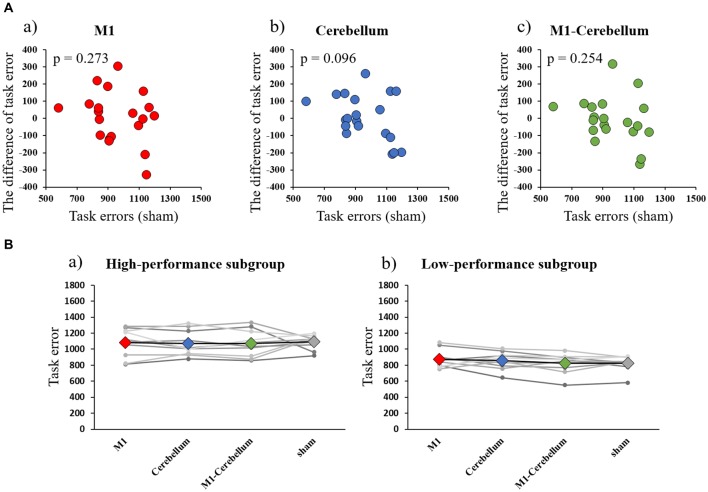
**(A)** Correlations between the difference in task error for each tACS condition and task errors during the sham condition (Experiment 2). **(a)** M1 condition. **(b)** Cerebellum condition. **(c)** M1-Cerebellum condition. **(B)** The mean values of task errors in beta tACS for each performance subgroup (Experiment 2). **(a)** High-performance subgroup. **(b)** Low-performance subgroup. tACS, transcranial alternating current stimulation; M1, primary motor cortex.

**Figure 5:**
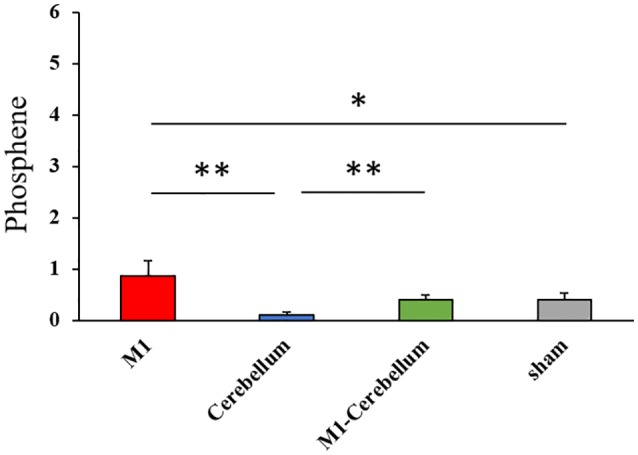
The intensity of phosphenes during beta tACS for each tACS condition. *p < 0.05, **p < 0.01. tACS, transcranial alternating current stimulation.

The mean values of task errors for each tACS condition were 969.4 ± 47.4 (M1 condition), 967.3 ± 44.0 (Cerebellum condition), 952.7 ± 45.7 (M1-Cerebellum condition) and 958.2 ± 34.9 (sham condition). There was no significant difference in the task error of sham condition between Experiments 1 and 2 by paired t-test (*P* = 0.202). A one-way repeated-measures ANOVA revealed no statistical difference (*F*_(1.878,35.685)_ = 0.188, *P* = 0.816, partial *η*^2^ = 0.010). No significant correlation between the task error during the sham condition and the difference in task error was found for M1 (*P* = 0.273), Cerebellum (*P* = 0.096) and M1-Cerebellum (*P* = 0.254) conditions (Figure 4A). Table 1 shows the mean values of task error for each tACS condition and subgroups. Figure 4B shows the task errors for each tACS condition in the two performance subgroups. A one-way repeated-measures ANOVA did not reveal a significant main effect in either the low-performance subgroup (*F*_(1.541,13.872)_ = 0.109, *P* = 0.848, partial *η*^2^ = 0.012) or the high-performance subgroup (*F*_(3,27)_ = 1.237, *P* = 0.316, partial *η*^2^ = 0.121). Figure 5 shows the intensity of the phosphenes the subjects reported experiencing (on a scale of 0–6) at each tACS condition. A one-way repeated-measures ANOVA revealed a significant main effect (*F*_(2.101,39.927)_ = 10.972, *P* < 0.001, partial *η*^2^ = 0.366). *Post hoc* analyses showed that the intensity of the phosphenes under the M1 condition was significantly greater than that under the Cerebellum condition (*P* = 0.001) and sham condition (*P* = 0.031), and that the intensity of the phosphenes under the M1-Cerebellum condition was significantly greater than that under the Cerebellum condition (*P* = 0.006).

## Discussion

This study investigated whether motor performance improved with gamma tACS over M1 and the cerebellar cortex region. The results demonstrated that gamma tACS over M1 and the contralateral cerebellar hemisphere did indeed improve motor performance for the subjects with lower motor performance. This effect was not observed with beta tACS, indicating that the improvement was dependent on the stimulation frequency.

### Effect of tACS Over M1

tACS applied to M1 at gamma and beta frequencies did not improve motor performance during stimulation. A previous study reported that, although tACS at 70 Hz on M1 and the shoulder increased motor velocity and motor acceleration during stimulation, the performance of a visuomotor tracking task did not improve (Moisa et al., 2016). It has also been reported that tACS at 20 Hz did not improve motor performance (Moisa et al., 2016). Our results are in line with these previous reports. Beta band activity has been shown to decrease prior to and during voluntary movement (Muthukumaraswamy, 2011) and to increase during sustained contraction (Brown and Marsden, 1998). In other words, the beta band activity might decrease during a visuomotor tracking task that requires constant adjustment of the contraction strength. Therefore, beta tACS condition may not have been effective in the present study. Not only beta tACS condition but also gamma tACS condition had no stimulatory effect. The previous study demonstrated that tACS at 80 Hz on the M1 and Cz areas improved the performance of a visuomotor tracking task during stimulation (Santarnecchi et al., 2017). In that study, the electrode was placed on Cz; the premotor cortex, supplementary motor cortex and primary somatosensory cortex were therefore located under this electrode. Because these areas are involved in the motor task requiring motor control (Rao et al., 1993; Shibasaki et al., 1993; Catalan et al., 1998), the improvement in motor performance observed in that study may be related not only to the M1 region but also to the simultaneous stimulation of multiple regions involved in the visuomotor tracking task. However, it is unknown whether simultaneous stimulation of the M1 region and Cz contributed to improving the performance, because the study did not investigate changes in motor performance when stimulation was applied to either the M1 region or Cz alone. In the present study, the motor performance during stimulation did not change when either gamma or beta tACS was applied at an intensity of 1 mA only to M1.

### Effect of tACS Over the Cerebellar Hemisphere

Gamma and beta tACS applied to the cerebellar hemisphere did not improve motor performance during stimulation. This study is the first report to investigate changes in motor performance during tACS on the cerebellar hemisphere region. Both M1 and the cerebellar hemisphere region are involved in tasks requiring motor control (Habas et al., 2004). The motor function of the fingers improved after applying tACS to the cerebellum, and an aftereffect of this has been demonstrated (Naro et al., 2017). We predicted that the effect of tACS on the cerebellum would be observed even during stimulation, but no such effect was found in this study. Our results show that motor performance during stimulation did not change when either gamma or beta tACS was applied only to the cerebellar hemisphere region.

### Effect of tACS Over M1 and the Cerebellar Hemisphere

Applying gamma tACS over M1 and the contralateral cerebellar hemisphere improved motor performance for subjects with low motor performance. This effect was not observed with beta tACS, demonstrating that the improvement was dependent on the stimulation frequency. A “binding theory” has been proposed, in which the neural populations excited in different cortical regions synchronize with the gamma band oscillation to strengthen the intercortical neural network (Lee et al., 2003). Yamamoto et al. (2014) demonstrated that high gamma synchrony of the hippocampus and entorhinal cortex contributed to the successful execution of spatial working memory. Hagiwara et al. (2010) showed that the activities of the primary and secondary somatosensory cortex were synchronized under gamma oscillations in the early stage of human somatosensory information processing. It has also been reported that the coherence of the gamma band increased between C3–C4, C3–Cz and C4–Cz during a typing movement task, suggesting that synchronization of the gamma band in the motor-related region is important for complicated movement tasks (Minc et al., 2010). The results of the present study may also involve the synchronous gamma band activity between the cortices. In this study, M1 and the cerebellar hemisphere region were stimulated simultaneously by gamma tACS, with the currents flowing through the two electrodes always in anti-phase. The time required for one cycle of the alternating current waveform when stimulated at a frequency of 70 Hz is approximately 14 ms. It is clear that it takes approximately 5–7 ms for the neurotransmission between M1 and the cerebellar hemisphere region because MEP measured by magnetic stimulation to M1 attenuates 5–7 ms after magnetic stimulation to the cerebellum (Naro et al., 2017). Thus, when the signal from the cerebellar cortex area was transmitted to M1 via the thalamus in the present study, taking approximately 5–7 ms, the current flowing to the electrode placed on M1 may have been in phase with the current flowing in the cerebellar cortex region. In other words, the functional synchronization between M1 and the cerebellar cortical area may have been enhanced. A previous study demonstrated a neural network via the thalamus between M1 and the cerebellar cortex region, showing that this neural network played an important role for performing exercise smoothly (Naro et al., 2017). Thus, it is possible that the task errors during the isometric force task in the present study decreased because the neural network between the cortices was strengthened by the simultaneous stimulation of M1 and the cerebellar cortex region by the gamma tACS. We did not observe an improvement in motor performance in the high-performance subgroup. This was probably the result of the ceiling effect.

### Influence of Phosphenes on Beta tACS

In Experiment 2, the greatest intensity of phosphenes was experienced in the M1 condition, and the least in the Cerebellum condition. This suggests that the electrode position is related to these results. In all three conditions except the Cerebellar condition, electrodes were attached to the M1 region; conversely, in the Cerebellum condition, no electrode was attached to the M1 region. A previous study reported that the intensity of phosphenes increased when electrodes were applied to the parietal or frontal lobe regions rather than to the occipital lobe region (Raco et al., 2014). This may be why the intensity of phosphenes was lowest in the Cerebellum condition in the present study. Under the sham condition, eight of the 20 subjects responded that they experienced mild phosphenes (scored as 1) at the start of stimulation. In the sham condition, an electrode was positioned on the left M1 and stimulated for 10 s (for fade in/out); the subjects therefore experienced mild phosphenes at the start of stimulation. However, although there were differences in the intensities of the phosphenes experienced in the different tACS conditions, the intensities were always low; we therefore believe that an isometric force task could be performed with the intensity of the phosphenes kept to a minimum.

## Limitations

There are some limitations to this study. First, the reference electrode positions are different between Experiments 1 and 2. We decided the reference electrode position to be on the right cheek based on previous studies (Im et al., 2012; Tseng et al., 2018). However, the reference electrode position was changed to the right shoulder in order to minimize the effect of phosphenes. Although the current density by tACS varies depending on the reference electrode position, it is unclear whether the current density is different between the right cheek and right shoulder (Mehta et al., 2015). The current density by transcranial direct current stimulation is the same whether the reference electrode position is the right cheek or the right shoulder (Im et al., 2012). Similarly, the current density by tACS might be the same whether the reference electrode position is the right cheek or the right shoulder. However, because the current density by tACS was not verified in this study, the difference in reference electrode position might have affected the stimulus effect. Second, because we did not measure changes in the frequency activity or the neural connectivity between the M1 and the cerebellar cortex areas during tACS, we cannot provide neurophysiological evidence, and further study is therefore needed. Nevertheless, the results of this study clearly showed that exercise performance improved with gamma tACS applied to both M1 and the cerebellar cortex region compared with when stimulating either cortex region alone. This is the first report on the influence of tACS on motor performance when applied to both M1 and the cerebellar cortex region.

## Conclusion

Gamma tACS over M1 and the contralateral cerebellar hemisphere improved motor performance in a visuomotor task for subjects with low motor performance. This effect was not observed with the M1 region alone, the cerebellar hemisphere alone or the beta tACS condition. These data support the hypothesis that strengthening the neural network between the cortices by gamma tACS over M1 and the cerebellar cortex region may improve the performance of a movement task requiring motor control.

## Author Contributions

HO and NO conceived the study, designed the experiments and performed interpretation of data. SK and SM conducted the experiments and performed the statistical analysis. SM wrote the manuscript. HO, KS, YI and MM provided feedback and edited the manuscript. All authors read and approved the final manuscript.

## Conflict of Interest Statement

The authors declare that the research was conducted in the absence of any commercial or financial relationships that could be construed as a potential conflict of interest.
